# Bystander Response and Out-of-Hospital Cardiac Arrest Outcomes (Bro. Study) in 3 Gulf Countries: Protocol for a Prospective, Observational, International Collaboration Study

**DOI:** 10.2196/58780

**Published:** 2024-11-12

**Authors:** Munawar Farooq, Mahmood Al Jufaili, Faisal K Hanjra, Shabbir Ahmad, Emad Hanna Dababneh, Omar Al Nahhas, Khalid Bashir

**Affiliations:** 1 Department of Internal Medicine, Emergency Medicine Section College of Medicine and Health Sciences United Arab Emirates University Al Ain United Arab Emirates; 2 Department of Emergency Medicine Tawam Hospital Al Ain United Arab Emirates; 3 Department of Emergency Medicine Sultan Qaboos University Hospital (SQUH) Muscat Oman; 4 Department of Emergency Medicine Sheikh Shakhbout Medical City Abu Dhabi United Arab Emirates; 5 Department of Emergency Medicine Hamad General Hospital Doha Qatar; 6 Life Support Center, Academic Affairs Tawam Hospital Al Ain United Arab Emirates; 7 Clinical Academic Education Qatar University Doha Qatar

**Keywords:** out-of-hospital cardiac arrest, cardiac arrest outcomes, bystander response, cardiopulmonary resuscitation, CPR, automated external defibrillator, AED, survival to discharge, emergency medical services, prehospital care, Utstein style

## Abstract

**Background:**

**:** Globally, there is significant variation in the out-of-hospital cardiac arrest (OHCA) survival rate. Early links in the chain of survival, including bystander cardiopulmonary resuscitation (CPR) and the use of an automated external defibrillator at the scene, are known to be of crucial importance, with strong evidence of increased survival rate with good neurological outcomes. The data from the Middle East are limited and report variable rates of bystander CPR and survival. It is crucial to get prospective, reliable data on bystander response in these regions to help plan interventions to improve bystander response and outcomes.

**Objective:**

This international collaborative study aims to describe the characteristics, including bystander interventions and outcomes, of OHCAs brought to hospitals enrolled in the study from Abu Dhabi, United Arab Emirates; Doha, Qatar; and Muscat, Oman. It also aims to describe the strength of the association between bystander response and OHCA outcomes, including the return of spontaneous circulation, survival to hospital admission, survival to discharge, and good neurological outcome at discharge in the local context of low bystander CPR rates.

**Methods:**

This multicenter, prospective, noninterventional observational study (Bro. Study) will be conducted at the emergency departments of 4 participating tertiary care hospitals in 3 countries. The data will be collected prospectively according to the Utstein style (a set of internationally accepted guidelines for uniform reporting of cardiac arrests) on demographic variables (age, sex, nationality, country, participating center, and comorbidities), peri–cardiac arrest variables (location, witnessed or not, bystander CPR, use of automated external defibrillator, time of emergency medical services arrival, initial rhythm, number of shocks, and time of prehospital CPR), and outcome variables (return of spontaneous circulation, survival to discharge, and neurological outcome at discharge and 3 months). Univariate and multivariate analysis with logistic regression models will be used to measure the strength of the association of bystander interventions with outcomes using SPSS (version 22).

**Results:**

Data collection began in November 2023 and will continue for 2 years, with publication expected by early 2026.

**Conclusions:**

Bystander response to an OHCA is critical to a favorable outcome. The reliable, baseline bystander CPR data will be a cornerstone in the team’s next planned projects, which are to qualitatively identify the barriers to bystander CPR, conduct a scoping review of community interventions in the Gulf and other Asian countries, and design and implement strategies to help improve the bystander CPR rate in the community.

**International Registered Report Identifier (IRRID):**

DERR1-10.2196/58780

## Introduction

### Background

Out-of-hospital cardiac arrest (OHCA) represents a substantial public health challenge, contributing significantly to morbidity and mortality across societies [1]. Timely and coordinated responses from the community and prehospital emergency medical services (EMS) are crucial in mitigating OHCA-related fatalities [2]. The early components of the chain of survival, comprising early recognition of cardiac arrest, prompt activation of EMS, provision of high-quality cardiopulmonary resuscitation (CPR), and timely defibrillation, are widely acknowledged as pivotal factors associated with increased rates of return of spontaneous circulation (ROSC) and improved neurological outcomes upon hospital discharge [2].

Notably, family members or bystanders assume a critical role in initiating this chain of survival by administering early and effective CPR. A systematic review and meta-analysis underscore the importance of swift delivery of evidence-based interventions by witnesses, with bystander CPR yielding a number needed to treat of 17 in areas with low baseline bystander CPR rates [3]. Studies show that bystander CPR can be associated with a 3-fold increase in survival with good neurological outcomes. Early use of an automated external defibrillator (AED) individually doubles the chance of survival and good neurological outcome [3,4]. Bystander CPR is also associated with higher rates of first shockable rhythm and higher survival rates, including survival with good neurological outcomes [4]. Enhanced care at pivotal junctures in the chain of survival is paramount, given the substantial patient volume before irreversible neurological damage occurs [5].

Discrepant survival rates following OHCA are documented globally, indicative of varying levels of effectiveness in community and prehospital EMS responses [6]. Such disparities are reflected in the variability of reported bystander CPR rates and AED use worldwide, with observed correlations between response efficacy and survival rates [7]. A recent study in Australia found lower rates of training and willingness to do CPR and use AED among migrants in Australia [8]. Regional studies have identified spatial disparities in bystander CPR rates, suggesting the potential for tailored CPR training strategies to address such discrepancies effectively [8].

A recent systematic review found that long-term survival and favorable neurological outcomes were lower in Asian and Middle Eastern countries than in European and North American countries [7]. Globally, there are examples of the focus on improvement in bystander CPR and AED use that improved all the relevant outcomes of OHCA [9]. Development of community-integrated response in Ireland over a period of 5 years increased the bystander CPR rate from 69% to 81%, improved the ROSC rate from 23% to 26%, and increased the survival to discharge from 6.3% to 7.2% with a cerebral performance score of 1 or 2 in most of them [10]. In the Pan-Asian Resuscitation Outcomes Study, implementing a dispatch CPR mechanism improved the rate and quality of bystander CPR, resulting in a measurable increase in survival with favorable neurological outcomes [11].

The Middle East and South Asia exhibit significantly lower OHCA survival rates and bystander CPR rates than global averages, with bystander CPR rates varying widely within the region [12,13]. One paper reported a bystander CPR rate of 30% [12], while another reported 5.1% [14]. The rate of AED use was reported to be less than 3% in Gulf Cooperation Council (GCC) countries. These variations underscore the complexity of OHCA management in the region, necessitating tailored interventions and further research to improve care and enhance survival rates.

While international literature underscores the critical importance of early CPR and defibrillation, a lack of uniform OHCA data reporting from the Middle East contributes to a limited understanding of regional OHCA dynamics.

Unique demographic, spatial, and cultural factors in the Middle East may influence bystander and community responses to OHCA, necessitating context-specific interventions. For example, in the United Arab Emirates (UAE) until 2019, there has been a lack of Good Samaritan laws to legally protect a layperson who attempts to provide first aid and resuscitation to an unconscious patient. Favorable laws have been introduced, but their favorable impact on community response is yet to be achieved and measured. Studies from the Middle East also report an alarmingly young median age of 51 years among patients with OHCA [15,16]. Furthermore, the absence of comprehensive in-hospital documentation practices aligned with Utstein guidelines in many Gulf countries underscores the region’s persistent data gaps surrounding OHCA.

In light of these challenges, our study aims to enhance the clinical documentation of OHCA cases in line with Utstein and cardiac arrest registry standards, facilitating comprehensive data collection from prehospital and hospital sources. By examining the association between bystander CPR and AED use with OHCA outcomes, including ROSC, survival to discharge, and favorable neurological outcomes, we seek to provide critical insights into local OHCA dynamics in the Middle East. These baseline data will inform future research endeavors to address barriers to early CPR in the community and implement targeted strategies to optimize OHCA response and outcomes. Given the current low baseline bystander response rates and OHCA survival outcomes, we anticipate that improving bystander CPR rates will lead to tangible enhancements in survival rates with favorable neurological outcomes upon discharge.

### Aims

#### Primary Outcome

The primary outcome is to describe the characteristics, including bystander interventions and outcomes, in OHCAs brought to hospitals enrolled in the study from Abu Dhabi, UAE; Doha, Qatar; and Muscat, Oman.

#### Secondary Outcome

The secondary outcome is to describe the strength of the association between bystander response and OHCA outcomes, including the ROSC, survival to hospital admission, survival to discharge, and good neurological outcome at discharge in the local context of low bystander CPR rates.

## Methods

### Study Design

This study (Bro. Study) adopts a prospective, observational, descriptive design conducted through international collaboration across 3 GCC countries.

### Setting

The international collaborative effort involves prospectively recording nontraumatic OHCA data upon presentation to emergency departments. Prehospital information from EMS and hospital records will be collected prospectively in the emergency department. Where feasible, information will also be gathered directly from prehospital records.

### Study Period

Data will be prospectively collected from November 1, 2023, to October 31, 2025. During this period, based on historical caseloads, the study is expected to include over 2000 cases of OHCA across the participating hospitals.

### Inclusion Criteria

The study will include all patients with nontraumatic cardiac arrest aged >18 years who are transported to participating emergency departments from non–health care facilities during the study period, wherein either bystanders or EMS provided prehospital resuscitation.

### Exclusion Criteria

Patients meeting the criteria for undeniable death as declared by EMS at the scene, where neither bystander nor EMS resuscitation was attempted and no resuscitation was performed in the emergency departments, will be excluded from the study.

### Data Collection Protocol

The emergency departments of Tawam Hospital, Al Ain, Abu Dhabi; Sheikh Shakhbout Medical City Abu Dhabi; Hamad General Hospital Doha; and Sultan Qaboos University Hospital Oman will collect data according to the Utstein style, a set of internationally accepted guidelines for uniform reporting of cardiac arrests. Ad hoc forms have been added to the existing electronic records to capture core and supplemental data per the International Liaison Committee on Resuscitation consensus guidelines [17]. Table 1 details the parameters to be collected. The details of the variables and response options for collecting data on OHCA are outlined in Multimedia Appendix 1.

**Table 1:** Key parameters.

Variables	Parameters
Demographic variable	Age Sex Nationality Country Presenting hospital Comorbidities
Peri–cardiac arrest variables	Location Witnessed status Bystander CPR^a^ AED^b^ use EMS^c^ arrival time Initial rhythm Prehospital shocks Prehospital CPR time
Post–cardiac arrest variables	ROSC^d^ Survival to discharge Neurological outcome at discharge Neurological outcome at 3 months

^a^CPR: cardiopulmonary resuscitation.

^b^AED: automated external defibrillator.

^c^EMS: emergency medical services.

^d^ROSC: return of spontaneous circulation.

### Prehospital Data

Prehospital resuscitation data will be prospectively collected using the Utstein-style international guideline data form from EMS. Only 1 center electronically transfers this data to hospital records; efforts are ongoing to implement this feature across all participating hospitals. A standardized handover sheet was designed for recording findings, which are promptly entered into electronic data following resuscitation. The parameters to be collected are detailed in Table 1.

### In-Hospital Data

In-hospital data on patients with OHCA will be systematically collected upon emergency department arrival using a report form designed for this study, adhering to Utstein guidelines. This form captures patient status (pulse, breathing, and cardiac rhythm), defibrillation details (number of shocks), use of mechanical CPR devices, advanced airway interventions (type specified), and vascular access types (intravenous, intraosseous, or central line) for medication administration (eg, epinephrine, atropine, amiodarone, lidocaine, bicarbonate, and dextrose).

### Outcome Data

Outcomes will be recorded prospectively, including ROSC, survival to discharge, and neurological status, using the Cerebral Performance Category scale at discharge. Residents involved in the study will collect this data following clear guidelines to ensure consistent and accurate entry. The same doctor who took the EMS handover and participated in the OHCA management will make electronic notes.

### Data Quality and Standardization

To ensure quality control and standardization across the 3 centers, personnel from 3 research teams will regularly review the collected data for consistency and accuracy, providing feedback to the participating centers. Regular meetings will align team members with study protocols and Utstein-style guidelines. Periodic audits will identify and rectify discrepancies or deviations, ensuring high data quality and uniformity across all participating centers. A full-time research assistant is recruited to look after these processes using the research grant fund.

### EMS Characteristics of Participating Hospitals

#### Doha, Qatar

The Hamad Medical Corporation Ambulance Service is Qatar’s sole ambulance provider, servicing a population of 3 million. Key units include Alpha (basic ambulance), Charlie (critical care), and Delta (supervisory). A hub-and-spoke model ensures rapid response, with a median EMS response time of 6-8 minutes [18].

#### Abu Dhabi, UAE

The Abu Dhabi Civil Defence Authority provides emergency services to 3.8 million residents. Units include basic (staffed by emergency medical responders and emergency medical technicians), advanced life support ambulances (staffed by advanced care paramedics and paramedics), and critical care ambulances (staffed by critical care paramedics).

#### Muscat, Oman

The Public Authority for Civil Defense and Ambulance, managed by the Royal Oman Police, oversees EMS. Ambulances are staffed with advanced emergency medical technicians and specialized drivers. The goal is a response time of 10 minutes in urban areas and 20 minutes in rural areas [19].

### Ethical Considerations

The study was approved by the Abu Dhabi Health Research and Technical Ethics Approval Committee on March 2, 2023, under the reference DOH/CVDC/2023/490 and by the Tawam Human Research Ethics Committee on May 2, 2023, under the reference KD/AJ/939.

The trial will follow the ethical guidelines established in the Declaration of Helsinki and the Belmont Report (1979).

## Results

The project successfully secured funding in 2023. Data collection commenced on November 1, 2023, and will be conducted prospectively over 2 years, concluding on October 31, 2025. The research findings will be published in a peer-reviewed academic journal, with the anticipated release in the first quarter of 2026.

## Discussion

### Expected Findings

This study anticipates a low incidence of bystander CPR and, correspondingly, very low use of AEDs within community settings in the Middle East. Given this context, favorable outcomes among patients with OHCA, even those who received bystander CPR, are expected to be limited. Such limitations may be attributed to delays in the initiation of CPR, thereby reducing its overall effectiveness. A critical barrier to addressing this issue is the lack of reliable and updated regional data, which complicates efforts to assess and improve cardiac arrest outcomes accurately. Previous research has reported a wide range of survival rates following OHCA, varying from 5.1% to 30%.

This study will focus on outcomes for patients who experienced witnessed cardiac arrests and received bystander CPR. Positive findings within this subgroup may suggest that increasing the prevalence and quality of bystander CPR, particularly in witnessed cases, could substantially enhance survival rates for OHCA, as supported by existing literature [9,10].

This study will analyze OHCA outcomes across 3 countries to provide insights for policy makers, guiding evidence-based policy development and community interventions [20].

### Future Plans

This investigation represents the initial phase of a 4-year, multiphased international collaborative research initiative to improve community health outcomes by examining bystander involvement in OHCA cases across 3 GCC nations. The goal of following sequential research is to implement strategies to enhance OHCA outcomes within the community.

1. Assess the current rates of bystander-administered CPR, AED use, and survival rates in the study regions compared to global benchmarks through a prospective, descriptive analysis of OHCA cases, which is the primary focus of this phase
2. Conduct a qualitative analysis of facilitators and barriers to bystander responses during OHCA incidents in the Al Ain region
3. Identify community-based interventions to increase bystander CPR rates in GCC countries, drawing on findings from the first 2 studies and a review of international literature on community interventions that improve OHCA outcomes in the GCC and Asia
4. Evaluate the effectiveness of community interventions by comparing bystander willingness, CPR rates, and OHCA survival rates against baseline rates established in the initial phase over 1 year
5. Establish a nationwide cardiac arrest registry in the study regions

### Limitations

Data collection will occur during the EMS handover to the resuscitation team. However, potential recall bias may arise among EMS personnel if pertinent information is not explicitly documented at the scene. Moreover, within the study region, EMS systems exhibit variability in response and documentation protocols, thereby influencing the quality and completeness of reported information. Consequently, missing data will inevitably arise, necessitating the use of complete case analysis and multiple imputations to address this issue. To overcome this limitation, robust guidance for handover documentation is provided to all the participating centers.

The prospective hospital-based design of the study means that sample size estimation depends on expected presentations to participating hospitals, which is potentially smaller than that in retrospective studies using registry data. While this design improves data reliability, it may limit the study’s ability to robustly measure secondary outcomes, such as establishing strong associations between variables and outcomes, particularly if AED use in the community is very low.

Direct access to prehospital records may not be possible in all participating centers. Currently, in 1 participating country (Qatar), prehospital records are directly uploaded to hospital records. In the other 2 countries, such integration is not yet complete. This might lead to variable access to prehospital records and, consequently, missing data. Prospective data collection at the time of handover will mitigate this limitation.

Patients who were declared dead at the scene by EMS have been excluded from the study; hence, subsequent study findings will not include outcomes for patients whose care is terminated in the field.

### Conclusions

This investigation marks the first international collaborative research effort among 3 neighboring Gulf countries. The study aims to produce up-to-date and reliable data on bystander response and its association with survival rates for OHCA within the Middle Eastern context. The findings will aid in planning and implementing community-based interventions to enhance outcomes, including establishing cardiac arrest registries.

## Appendix

Multimedia Appendix 1 Out-of-hospital cardiac arrest variables and response options.

## Abbreviations

AED: automated external defibrillator
CPR: cardiopulmonary resuscitation
EMS: emergency medical services
GCC: Gulf Cooperation Council
OHCA: out-of-hospital cardiac arrest
ROSC: return of spontaneous circulation
UAE: United Arab Emirates

## Data Availability

The datasets generated and analyzed during the study will be available from the corresponding author (MF) upon reasonable request.
