# Supplementary material for: Bystander Response and Out-of-Hospital Cardiac Arrest Outcomes (Bro. Study) in 3 Gulf Countries: Protocol for a Prospective, Observational, International Collaboration Study
Source: JMIR Res Protoc. 2024 Nov 12;13:e58780. doi: 10.2196/58780 (PMC11599888; doi:10.2196/58780)
Supplement: Multimedia Appendix 1 [file resprot_v13i1e58780_app1.pdf]

Appendix 1: Out-of-Hospital Cardiac Arrest Variables and Response Options.

Category	*Data Heading*	*Response Options*
Demographics	Age	
	Sex	
	Nationality	
	Country	
	Presenting Hospital	
	Co-morbidities	
Mode of Transportation	The patient was brought in by	- Ambulance<br>- ALS/Air<br>- ALS/Ambulance<br>- BLS/Ambulance<br>- Carried<br>- Helicopter<br>- Police<br>- Private vehicle<br>- Walking<br>- Wheelchair<br>- Other
Incident Information	Location Type	- Home/Residence<br>- Healthcare Facility<br>- EMS/Private ambulance<br>- Public/Commercial building<br>- Street/Highway<br>- Industrial/Workplace<br>- Transport centers<br>- Nursing home<br>- Sport/recreation place<br>- Other
Patient Information	Past medical history	Available on Cerner
	Ability to perform daily activities independently	- Yes<br>- No<br>- Unknown

	-Patient supported by any ventricular assist device	- Yes<br>- No<br>- Unknown
	- Patient has internal/external Cardioverter-defibrillator	- Yes<br>- No<br>- Unknown
Prehospital Event and Resus Info	Estimated time of arrest	- Known (Insert date/time)<br>- Unknown
	EMS arrival time	- Known (Insert date/time)<br>- Unknown
	Arrest Witnessed by	- Not witnessed<br>- First responder<br>- EMS/Private Ambulance<br>- Bystander (Family/Healthcare Provider/Layperson)
	Bystander CPR	- Yes<br>- No<br>- NA
	Bystander AED Applied	- Yes<br>- No<br>- NA
	First CPR initiated by	- No CPR initiated<br>- First responder<br>- Ambulance Crew<br>- Bystander (Family/Healthcare provider/Layperson)
	Date & Time of CPR Started by EMS/Private ambulance	Specify date DD-MM-YYYY<br>specify time HH (24-hour format)
	Resuscitation Attempted by EMS	- Yes<br>- No
	First Arrest Rhythm	- VF<br>- VT<br>- Asystole<br>- PEA<br>- Unknown

	Prehospital defibrillation	- Yes (Insert time of the first shock, number of shocks)<br>- No<br>- Unknown
	Defibrillation performed by	- NA<br>- First responder<br>- Ambulance crew<br>- Bystander (Family/Healthcare provider/Layperson)
	Mechanical CPR device used by EMS/private ambulance	- Yes<br>- No
	Prehospital advanced airway	- Yes (Specify ETT/Surgical airway/SGAD)<br>- No
	Prehospital drug administration	- Yes<br>- No<br>_If yes specify the drug (Epinephrine/Amiodarone/Bicarbonate/Lidocaine/Dextrose/Other)<br>Specify the number of Epi given.
	ROSC at Scene/En-route	- Yes<br>- No
	CPR discontinued at the scene	-Yes<br>-No<br>-If yes, specify the reason<br>(DNAR, Medical control order, ROSC, Obvious signs of death)
EMS Disposition	Final Status at the scene	- Conveyed to ED<br>- Pronounced dead at the scene
	Cause of Arrest	- Trauma<br>- non-Trauma<br>If non-trauma, please specify (Presumed cardiac aetiology/Electrocution/Drug overdose/Respiratory/Asphyxia/Drowning/Other)
	Patient Status at ED	Ongoing resuscitation<br>ROSC<br>Brought in dead

ED Resuscitation Information	Patient Status on arrival at ED	-Insert Date/Time on arrival at ED<br>- Pulse: Yes/No<br>- Breathing: Yes/No
	Cardiac rhythm on arrival at ED	- VF<br>- VT<br>- Bradycardia<br>- Asystole<br>- PEA<br>- Sinus/Other perfusing rhythm
	ED defibrillation performed	- Yes (Insert number of shocks)<br>- No
	Mechanical CPR device used	- Yes<br>- No
	Advanced airway	- Yes (ETT/Surgical airway/SGAD)<br>- No
	Vascular access type	- IV<br>- IO<br>- Central line<br>- Endotracheal
	Drug administration	- Yes<br>- No<br>If yes Specify drugs<br>(Epinephrine/Amiodarone/Bicarbonate/Lidocaine/Dextrose/Other)<br>Specify times of Epi given
	Arterial Line	- Yes<br>- No
	VBG/ABG during resuscitation	- Yes<br>- No
	POCUS during Resuscitation	- Yes (Specify findings)<br>- No
	ROSC	- Yes<br>- No
	ECMO therapy initiated	- Yes<br>- No
	PCI Activated Score	Specify

	Coronary reperfusion attempted	- Yes (Thrombolysis/PCI/CABG)<br>- No
	Cause of arrest	- Trauma<br>- Non-trauma (Presumed cardiac aetiology/Respiratory/Asphyxia/Electrocution/Drowning/Drug overdose/Other)
Outcome Data	Outcome of patient	- Admitted (ICU/CCU/Other)<br>- Died in ED<br>- Transferred to another hospital<br>- Died in hospital (Insert time of death, cause of death, resuscitation status)<br>- Survived and discharged from hospital
	Discharge Destination	Specify
	Neurological status at discharge	Specify (CEREBRAL PERFORMANCE SCORE)
	Length of stay in ICU	Specify (Hours)
	Total length of stay in hospital	Specify (Hours)
	Neurological status at three months	Specify (CEREBRAL PERFORMANCE SCORE)
